# Coronary Stenosis and Cardiogenic Shock Secondary to Aortitis Following Aortic Root Support Procedure

**DOI:** 10.1016/j.jaccas.2024.102313

**Published:** 2024-03-22

**Authors:** Alexander Carpenter, Stephanie Connaire, Nitin Chandra Mohan, Stephanie L. Curtis, Serban C. Stoica, Massimo Caputo, Julian W. Strange

**Affiliations:** Bristol Heart Institute, Bristol, United Kingdom

**Keywords:** aortitis, exostent, Marfan, PEARS

## Abstract

A woman with recent personalized external aortic root support implant presented in cardiogenic shock with bilateral coronary ostial occlusion and aortic inflammation requiring emergency coronary angioplasty. Subsequent computed tomography with positron emission tomography scanning demonstrated aortitis with extensive inflammation adjacent to the personalized external aortic root support mesh, the first report of this important complication.

## Graphical abstract

**Figure:**
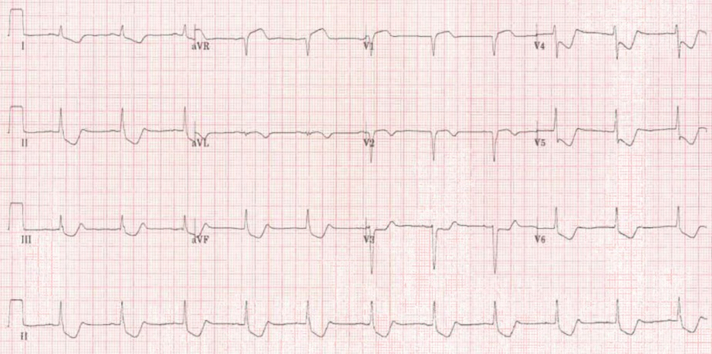

## Introduction

Personalized external aortic root support (PEARS) is a prophylactic surgical procedure where a custom-designed three-dimensionally printed sleeve of medical grade mesh is used to externally support the aorta. The primary indication for a PEARS procedure is to stabilize aortic dilatation in Marfan syndrome and other similar heritable thoracic aortopathies, decreasing the associated risk of aortic dissection and rupture. It is emerging as an attractive alternative to conventional surgical options, including composite aortic root replacement or valve-sparing aortic root replacement. PEARS can usually be performed off cardiopulmonary bypass, does not require reimplantation of the coronary arteries, and preserves the native aortic valve and endothelium.

**Learning Objectives**

• To understand the recent trend toward the personalized external aortic root support procedure as an alternative to surgical intervention to reduce the incidence of aortic dissection or rupture in Marfan syndrome.
• To appreciate the relevance of coronary complications that may occur as the result of such a procedure and the need for urgent assessment and/or intervention.
• To understand that such grafts may incur a postoperative inflammatory response.
• To understand that counselling patients regarding this prophylactic procedure may be more nuanced than originally thought.

The innovative technique shows promise as a safe and effective treatment^1^ with good long-term outcomes,^2^ although to date a randomized controlled trial is lacking. In 2022, the UK National Institute for Health and Care Excellence published its interventional procedures guidance on use of PEARS, concluding that there was sufficient evidence on the short-term safety and efficacy of the procedure, but limited long-term data.^3^

## History of Presentation and Past Medical History

We present the case of a 29-year-old woman with Marfan syndrome and a family history of aortic dissection. She had severe mitral regurgitation secondary to bileaflet mitral valve prolapse with a dilated left ventricle (LV) as a primary indication for surgery. In addition, she had a small secundum atrial septal defect and a mildly dilated aortic root measuring 38 mm at the sinus of Valsalva with progressive growth. After discussion in the multidisciplinary team meeting and fully informed discussion with the patient, she underwent surgical mitral valve repair with atrial septal defect closure and placement of an ExoVasc PEARS exostent, to decrease her future risk of aortic dissection. The use of the exostent compared with conventional repair was felt to offer a solution that could reduce cross-clamp time significantly.

The operation was undertaken successfully without any immediate complications, with a cross-clamp time of 1 hour and 51 minutes, and total bypass time of 3 hours and 7 minutes. Briefly, the aortic root was dissected off, and then on, bypass. The mitral procedure was completed initially. With the heart reperfused, the exostent was wrapped around the aortic root on bypass and a standard size was used, because there was no aortic insufficiency. The graft was fixed around the root in the standard fashion, ensuring sure that the hem sits inferiorly on the external ventriculoarterial junction. There were no concerns about coronary ischemia in the early postoperative phase in terms of clinical picture, electrocardiogram (ECG) changes, or on echocardiography.

She developed a fever and cough in the early postoperative period and was treated for a lower respiratory tract infection with oral antibiotics. Three weeks later, she was examined in the outpatient clinic and complained of back and shoulder discomfort. Transthoracic echocardiography revealed a significant pericardial effusion with a maximal diameter of 4 cm adjacent to the LV and features of hemodynamic compromise. She was, therefore, admitted for pericardiocentesis. Serosanguinous pericardial fluid was sent to the laboratory for microscopy and culture and there was no evidence of infection. She was discharged 2 days later with a prescription for oral ibuprofen.

Four months after the initial operation, she presented to the hospital as an emergency at night with chest pain and feeling generally unwell. On presentation to the emergency department, she reported a 7-hour history of persistent central crushing chest pain, radiating to her left arm and associated with nausea and feeling clammy. She appeared unwell, but with unremarkable physiological observations, including a heart rate of 72 beats/min, respiratory rate of 17 breaths/min, blood pressure (BP) of 113/82 mm Hg, and oxygen saturations of 98% on room air. She was afebrile with a temperature of 36.4°C.

## Differential Diagnosis

The differential diagnosis included aortic dissection, pulmonary embolism, and coronary ischemia.

## Investigations

Blood tests on arrival were largely unremarkable. Her C-reactive protein was 2 mg/L (reference value <6 mg/L). High-sensitivity troponin T was mildly elevated at 42 ng/L (reference value <14 ng/L).

**Figure 1:**
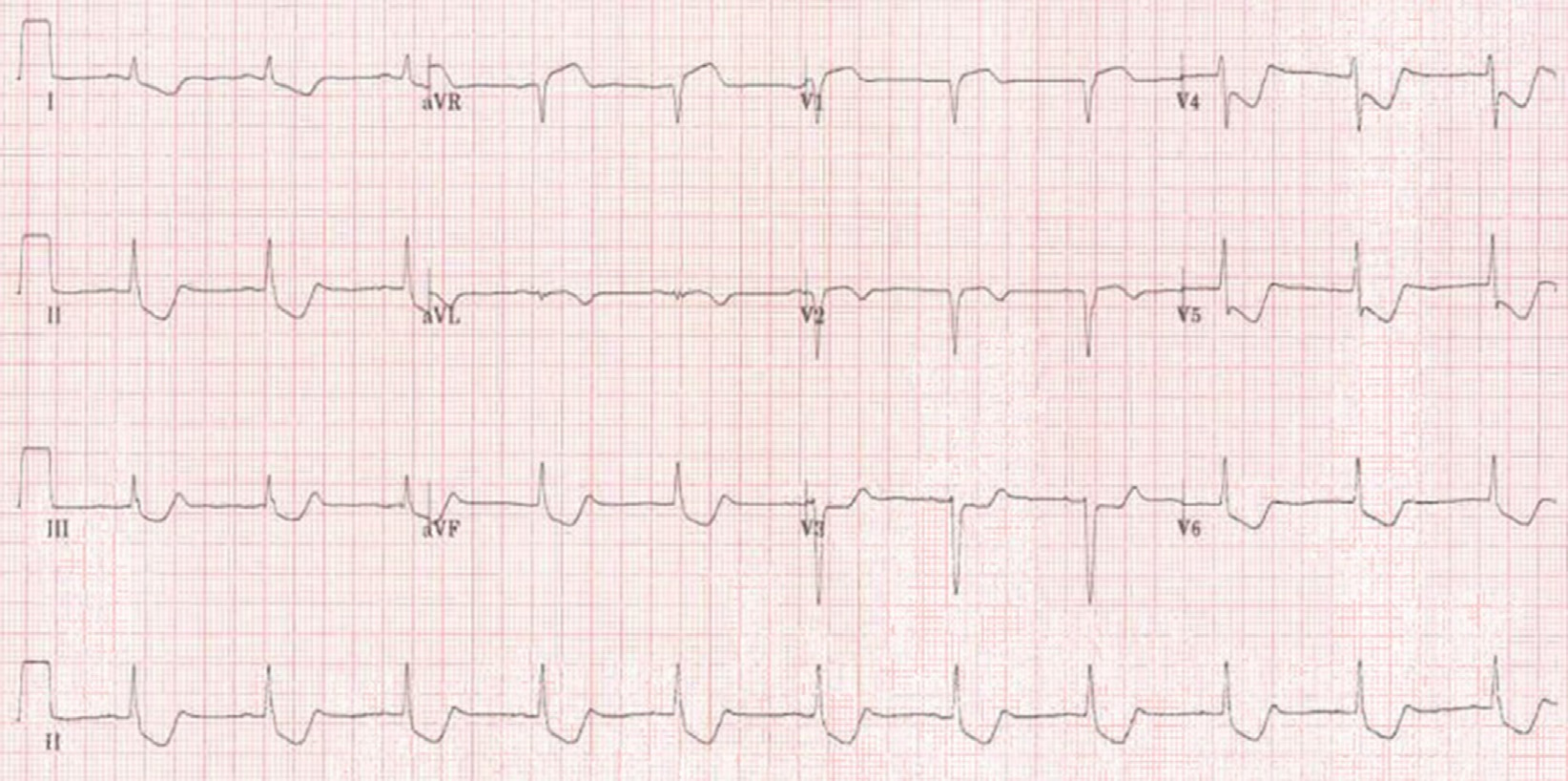
12-Lead ECG Obtained at Presentation Demonstrating Profound Cardiac Ischemia in a Left Main Stem Coronary Artery Configuration ECG = electrocardiography.

A 12-lead ECG revealed sinus rhythm with profound ischemia in a left main stem configuration (Figure 1). Bedside echocardiography was undertaken. No pericardial effusion was seen, but LV systolic function was severely impaired with akinesis of all mid and apical segments.

**Figure 2:**
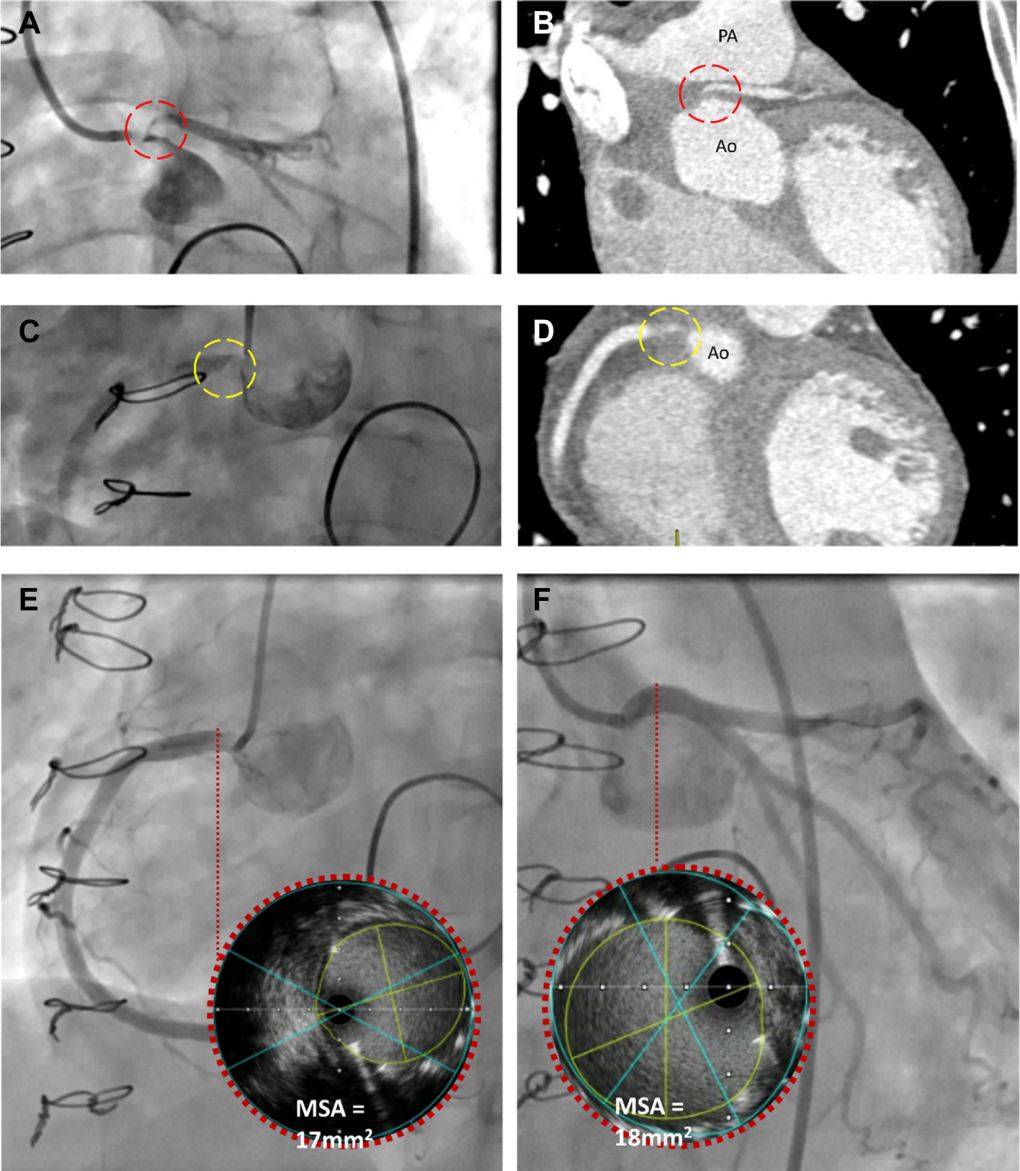
Severe Ostial Stenosis (A and C) Severe ostial stenosis of the left (red circle) and right coronary ostia (yellow circle) on invasive coronary angiography. (B and D) Respective findings on reconstructed computed tomographic coronary angiography. (E and F) Final angiographic result after successful deployment of drug eluting stents to the right (E) and left (F) coronary ostia with corresponding IVUS frames of MSA showing satisfactory deployment. Ao = aorta; IVUS = intravascular ultrasound; MSA = minimum stent area; PA = pulmonary artery.

An urgent gated computed tomography (CT) scan of the thorax was arranged to assess for aortic dissection. This reported subtotal occlusion of both coronary ostia, likely secondary to extensive aortitis seen adjacent to the PEARS exostent (Figures 2A and 2C). At this stage, the patient had become hypotensive with a systolic BP of 70 mm Hg, refractory to intravenous fluid therapy. A repeat ECG demonstrated worsening ischemic changes.

## Management

The patient was loaded with aspirin and ticagrelor and underwent emergency coronary angiography in the presence of senior cardiac surgical and cardiac anesthetic colleagues with left ventricular assist device (Impella CP) and cardiopulmonary bypass support on standby. On cannulation of the left coronary ostium, the systolic BP decreased to 40 mm Hg. Angiography revealed a subtotally occluded ostium with minimal flow (Figures 2B and 2D). This lesion was crossed with a wire and subsequently treated with balloon angioplasty and placement of a Synergy Megatron drug-eluting stent, chosen for increased radial strength. Cannulation of the right coronary system revealed a subtotally occluded ostium which was also stented. The stent was optimized using intravascular ultrasound examination; a good angiographic and intravascular ultrasound result was obtained, with TIMI flow grade of 3. Hemodynamic and ECG parameters resolved, and the chest pain settled (Figures 2E and 2F).

## Discussion

We contacted the ExoVasc PEARS manufacturer, who reported that only a small number of cases of coronary complications had been encountered during surgery or immediately postoperatively, but that this type of clinical picture several months postoperatively had not been seen previously.

The PEARS and AVIATOR registry data no not report this type of complication in 159 patients who have had an exostent implanted.^2^ Kočková et al^4^ reported an increased incidence of postoperative inflammatory response after PEARS procedure in comparison with that seen after the standard surgical approach. The PEARS group experienced significantly higher rates of postoperative raised C-reactive protein and white cell count, as well as early and late fever and pericarditis. Nemec et al^5^ reported adverse events for 317 patients undergoing PEARS in 25 surgical centres in 9 countries between April 2004 and March 2020. These largely occurred owing to intraoperative coronary artery injury. There were no reports of an excessive early or late inflammatory response in any of the patients.

## Follow-up

The patient was admitted to the coronary care unit after the procedure and remained pain free with improvement of ischemic changes on subsequent 12-lead ECGs. Her repeat troponin T was 1,266 ng/L and subsequently peaked at 2,194 ng/L.

**Figure 3:**
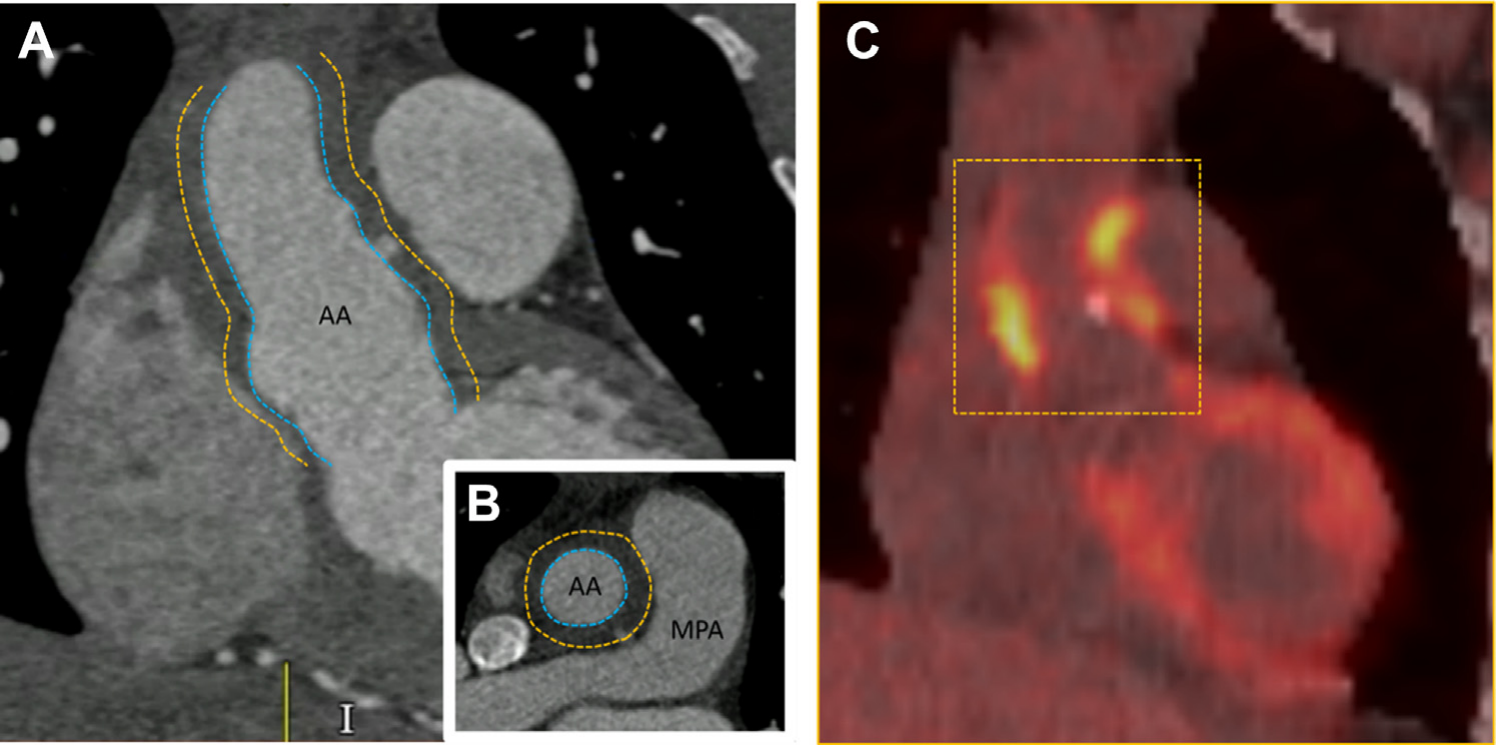
▪▪▪ (A and B) Coronal (A) and axial (B) CT images of extensive soft tissue thickening seen in the aortic root and ascending aorta (true lumen = blue dotted lines; extent of thickening = orange dotted lines). (C) PET-CT images of the corresponding section of ascending aorta with evidence of active irregular soft tissue (orange dotted box) consistent with extensive inflammation. AA = ascending aorta; CT = computed tomography; MPA = main pulmonary artery; PET = positron emission tomography.

Cardiac magnetic resonance revealed a mildly dilated and moderately impaired LV globally, with an ejection fraction of 44%, and inflammation around the ascending aorta, as well as a large mobile thrombus in the right atrial appendage. CT with positron emission tomography scanning described irregular active soft tissue around the ascending aorta consistent with extensive inflammation (Figure 3).

The multidisciplinary team felt that this likely represented a form of graft fever aortitis secondary to PEARS implantation. The right atrial appendage thrombus abutted an area of inflamed myocardium adjacent to the inflamed aorta; hence, the likely etiology of thrombus formation. Anticoagulation was initiated with apixaban.

The patient was commenced on heart failure therapy. A repeat echocardiogram 4 weeks later showed normalized LV systolic function. Further blood tests to exclude an underlying autoinflammatory or infective cause were negative. Erythrocyte sedimentation rate (22 mm/h) and anticardiolipin IgG (38.5 GPL U/mL) were mildly elevated.

A decision was made by the multidisciplinary team to start colchicine at a dose of 500 μg twice daily for its anti-inflammatory properties. A CT with positron emission tomography was repeated 5 weeks after the first scan, showing less extensive inflammation with reduced signal intensity.

The patient remains under close imaging surveillance but is clinically well.

## Conclusions

Much attention has been give to the advantages of PEARS over conventional valve-sparing aortic root replacement for Marfan syndrome. Registry data have focused on overall survival, freedom from redo surgery, and perioperative complications such as bleeding and coronary damage. We present a case where the postimplantation inflammatory reponse after PEARS was dramatic and life threatening. More importantly, it occurred several months after the operation in a well patient, without warning. This represents the first report of a serious and important complication of PEARS implantation, with extensive graft-adjacent aortitis causing life-threatening coronary compromise. Postoperative cross-sectional imaging after 3 to 6 months, as already undertaken after conventional aortic repair or replacement, might be advisable to exclude this important complication. More research is needed into the postimplantation response and how to monitor it after PEARS. Clinicians need to be aware of the possibility of this complication occurring after PEARS, when counselling patients regarding this prophylactic procedure.

## Funding Support and Author Disclosures

The authors have reported that they have no relationships relevant to the contents of this paper to disclose.
